# Diurnal Variations of Summer Precipitation Linking to the Topographical Conditions over the Beijing-Tianjin-Hebei Region

**DOI:** 10.1038/s41598-020-65743-1

**Published:** 2020-06-16

**Authors:** Ziyi Song, Jingyong Zhang

**Affiliations:** 1 Key Laboratory of Meteorological Disaster, Ministry of Education (KLME)/Collaborative Innovation Center on Forecast and Evaluation of Meteorological Disasters (CIC-FEMD), Nanjing University of Information Science and Technology, Nanjing, 210044 China; 2 Center for Monsoon System Research, Institute of Atmospheric Physics, Chinese Academy of Sciences, Beijing, 100029 China; 3 College of Earth and Planetary Sciences, University of Chinese Academy of Sciences, Beijing, 100049 China

**Keywords:** Climate sciences, Hydrology

## Abstract

The Beijing-Tianjin-Hebei (BTH) region of above 110 million people, located in North China, is confronted with high risks of precipitation-related disasters during the summer. Efforts to better understand diurnal variation characteristics of summer precipitation and associated physical driving processes are of vital importance to accurate forecast of short-time precipitation. Based on hourly gridded precipitation data at a fine resolution of 0.1° newly developed by China Meteorological Administration (CMA), we investigate diurnal variations of summer precipitation and their linkages with the topographical conditions in the BTH region for the period of 2008–2018. Summer precipitation amounts are shown to nonlinearly change with the topographical height, the largest values occurring at the altitudes of around 350 m in the BTH region. As a whole, diurnal variation of summer mean precipitation in the BTH region exhibits an S-shape structure with the peak appearing around 20:00 LST. While the mountainous precipitation largely triggers the precipitation peak with contribution from coastal and plain areas, the large precipitation in early morning is dominated by the precipitation over coastal and plain areas. Heavy and very heavy precipitation frequencies generally decrease with topographical height while light precipitation frequency increases in a nonlinear manner. The physical processes explaining the tight precipitation-topography linkages are also discussed. Our findings are expected to provide useful information for the improvement of short-time precipitation forecast over the BTH region.

## Introduction

Extreme short-time precipitation events frequently caused devastating impacts on ecosystems and human society worldwide^1–4^. These extreme events are closely tied to diurnal precipitation cycle primarily driven by solar radiation via its impacts on the interlinked thermodynamic and dynamic processes in the earth’s atmosphere^5–7^. Although the great significance has been widely recognized, understanding diurnal precipitation variations and associated complex physical mechanisms across various space scales is still very challenging.

Diurnal precipitation variations have been demonstrated to be remarkable globally, and the peaks vary with region and season^8–10^. For underlying physical processes, previous studies found that topographical conditions such as “mountain-valley wind” effect^11,12^ and windward slope effect^13,14^, land-sea distribution^15^, urbanizations^16,17^, atmospheric circulation background^18–20^ and other factors have important roles to play in driving diurnal precipitation variations over different regions. For example, due to the “mountain-valley wind” effect, the peak time of diurnal precipitation cycle occurs distinctly in mountain and valley areas^11^.

Previous studies have shown that there are strong spatial and temporal variations in the daily cycle of precipitation over China^7,21–27^, due to the diverse land surface characteristics^10^, complex topographical conditions^10–14^, land-sea distributions^15^, monsoon evolution^23,24^, regionally different atmospheric circulation among others^18–20^. For example, Yu *et al*.^21^ revealed that the peaks of diurnal precipitation cycle in warm-season can occur during the morning, afternoon and night, depending on specific regions. In general, studies on diurnal precipitation characteristics and their causes over China are mainly based on the hourly or sub-daily data from relatively limited stations, typically several hundred stations across China.

The Beijing-Tianjin-Hebei (BTH) region of North China is home to more than 110 million people, and is severely influenced by frequent heavy precipitation events during the summer in recent years. A series of studies have addressed short-time precipitation variations and daily precipitation extremes, and the associated physical causes over the BTH region, in particular Beijing^14,17,28–31^. For example, Yang *et al*.^28^ demonstrated that summer rainfall amount in Beijing is largely controlled by 1–3 h duration heavy rainfall events. Currently, diurnal characteristics of summer precipitation and underlying driving processes remain insufficiently investigated, partly due to the limitations of available observational data. Recently, a gridded hourly precipitation dataset of China at a resolution of 0.1° is developed by China Meteorological Administration (CMA), based on the observations of 30000–40000 automatic meteorological stations across China and the CMORPH^32^ (Climate Prediction Center(CPC) morphing technique) precipitation data provided by the National Oceanic and Atmospheric Administration (NOAA). This study aims to use the newly emerging fine-resolution dataset to improve our understanding of diurnal precipitation variations during the summer and how they depend on the complex topographical conditions over the BTH region for the period of 2008–2018.

## Results

### Diurnal variation characteristics

**Figure 1:**
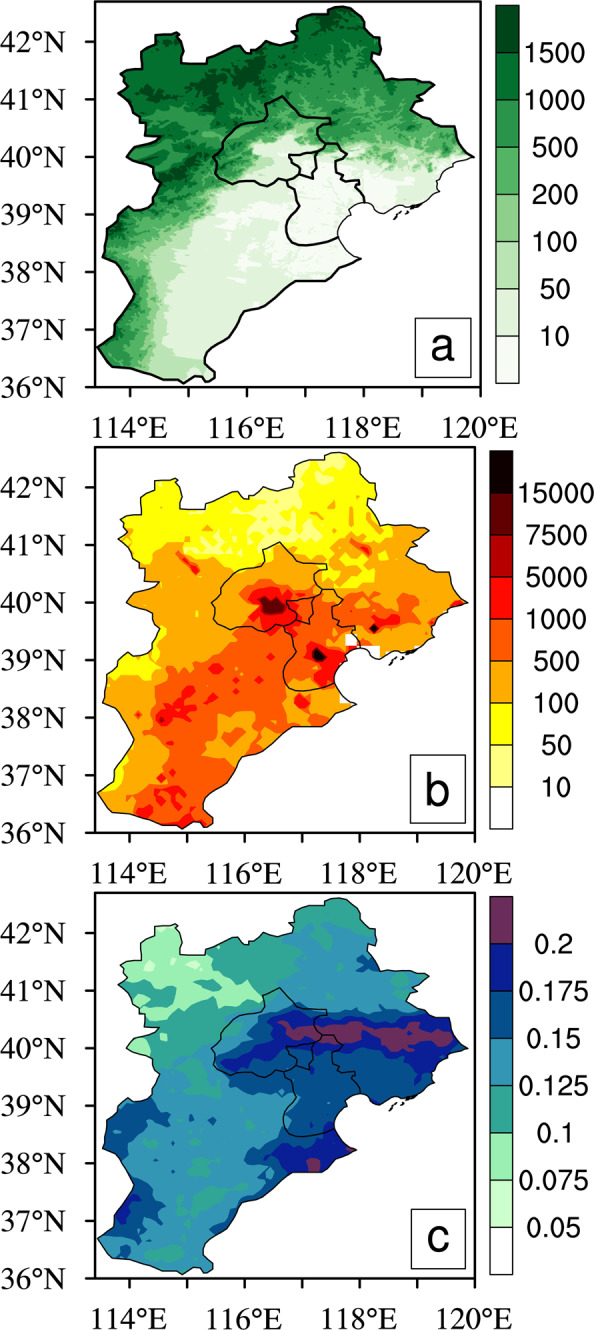
(**a**) Topographical height (units: m), (**b**) Population density (units: *people*/*km*^2^), and (**c**) Climatic pattern of summer precipitation from 2008 to 2018 (units: *mm*/*h*) in the BTH region. Figure was produced using NCL V6.4.0 (http://www.ncl.ucar.edu/).

The BTH region is located in the eastern part of China with a complex topography, which gradually decreases from northwest to southeast (Fig. 1a). In general, the coastal and plain areas of the BTH region are densely populated and highly urbanized (Fig. 1b). The climatic pattern of summer precipitation in the region for 2008–2018 shows that precipitation amounts generally exhibit an obvious gradient from northwest to southeast, and the precipitation amounts over mountain slopes, urban areas and coastal areas are much larger than those over the mountain areas (Fig. 1c). Over the BTH region, the maximum precipitation mainly occurs over the south slope of the Yanshan Mountain.

**Figure 2:**
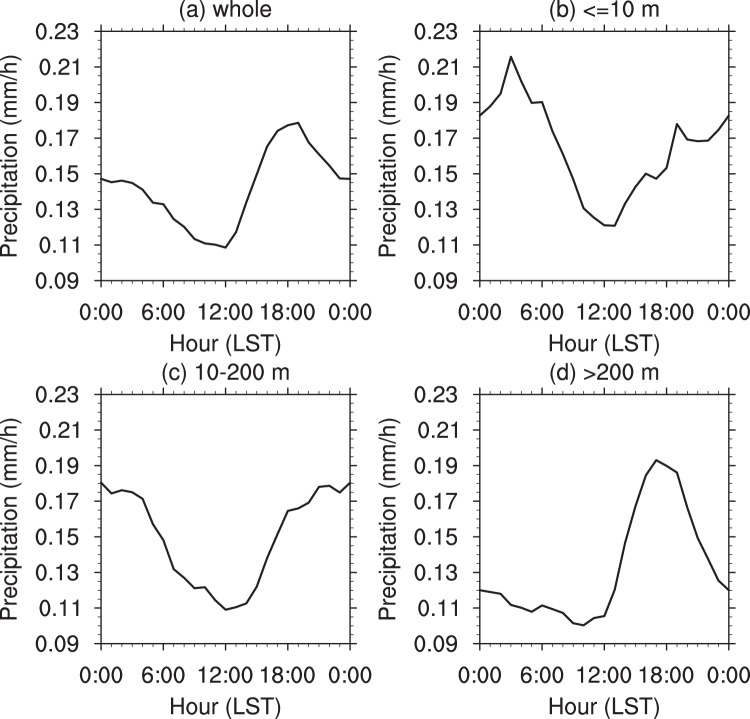
Regionally averaged diurnal variations of summer precipitation in the BTH region from 2008 to 2018 (units: *mm*/*h*): (**a**) the whole region, (**b**) the coastal areas below 10 m, (**c**) the plain areas from 10 to 200 m, (**d**) the mountainous areas above 200 m.

As a whole, diurnal variation of summer mean precipitation generally shows an S-shape curve in the BTH region (Fig. 2a): the value is nearly 0.15 mm/h during around 0:00–03:00 LST (local standard time), then gradually declines to the lowest at the noon, and after that increases rapidly to the peak around 20:00 LST. According to the percentage of hourly precipitation to 24-hour cumulative precipitation (see Supplementary Fig. S1), the temporal distributions of precipitation vary with the location. Summer precipitation in the northwestern mountainous areas of the BTH region mainly occurs from 14:00 to 20:00 LST, whereas the precipitation events from the evening to early morning dominate over southeastern plain and coastal areas.

We further examine diurnal precipitation cycles averaged over coastal areas (<10 m), plain areas (10–200 m) and mountainous areas (>200 m) of the BTH region (Fig. 2b–d). Diurnal precipitation characteristics are markedly distinct over three types of the topography. In the coastal areas (Fig. 2b), the precipitation decreases rapidly from early morning to noon and reaches a high value around 20:00 LST, then after a slight decline it rises again, reaching its peak around 3:00 LST, and subsequently decreases again to the lowest value around 12:00 LST. In comparison, the highest precipitation value at around 3:00 LST is about twice of the lowest value at around12:00 LST. The plain areas (Fig. 2c) are the transitional zones between coastal and mountainous areas, and have high precipitation with similar magnitude from 18:00 LST in the evening to 5:00 LST in the morning; there is a V-shape distribution during 5:00–18:00 LST with the lowest value occurring at around 12:00 LST. The precipitation in mountainous areas (Fig. 2d) has obvious diurnal variations with low values from early morning to noon, and then rapidly increases from afternoon to early evening, peaking at about 17:00 LST.

Diurnal precipitation variations over coastal, plain and mountainous areas play different roles in shaping diurnal precipitation cycle averaged over the whole BTH region. The peak around 20:00 LST over the BTH region is largely induced by the precipitation over mountainous areas overlapped with the contribution from coastal and plain areas. In addition, high precipitation in early morning is mainly contributed by the precipitation over the coastal and plain areas.

**Figure 3:**
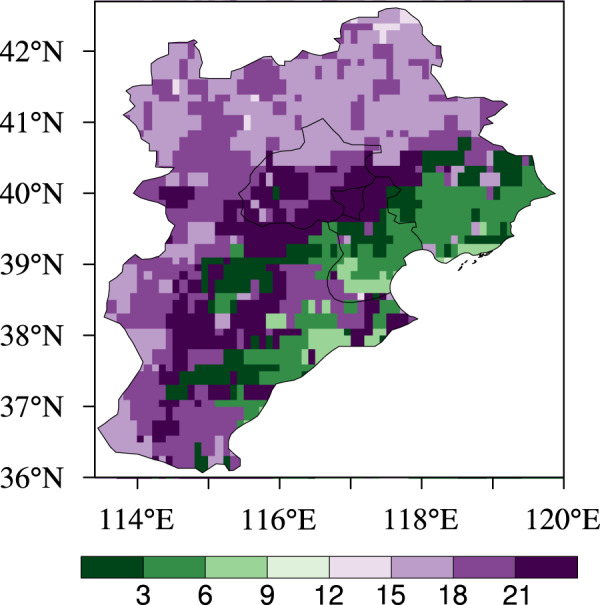
Distribution of peak precipitation period in the daily cycle of summer precipitation in the BTH averaged over the period 2008–2018. Figure was produced using NCL V6.4.0 (http://www.ncl.ucar.edu/).

There are remarkable spatial differences in diurnal precipitation peaks among three types of topography over the BTH region (Fig. 3). Specifically, the precipitation peak period in mountainous areas mainly occurs from 15:00 to 21:00 LST; for the northern plain areas, the period of precipitation peak appears from 21:00 to 0:00 LST, while the main peak period is from 21:00 to 6:00 LST over the southern plain areas; in the coastal areas, the precipitation peak period mainly exists from 3:00 to 9:00 LST.

### Relationship between precipitation and topographical conditions

**Figure 4:**
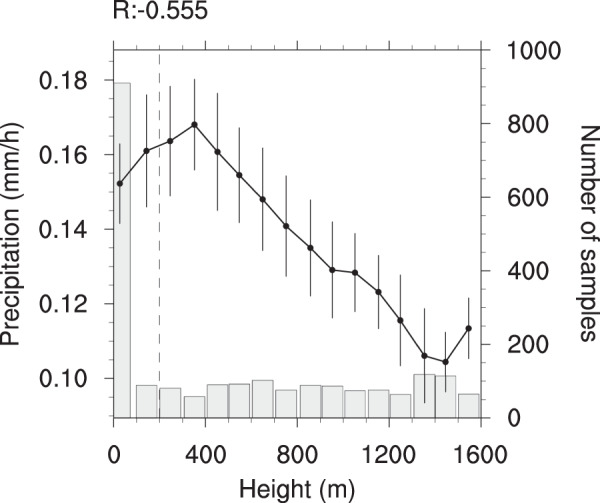
The linkage of summer precipitation to topographical height (below 1600 m) in the BTH region for 2008–2018. The line represents summer precipitation variations with topographical height (binned at an interval of 100 m), and the error bars are also shown (±0.5 standard deviation). The vertical blue dotted line in the map represents the boundary line between the plain and mountainous areas, and the column chart shows the number of samples in the corresponding set.

Figure 4 shows that summer precipitation is closely associated with the topographical conditions in the BTH region for 2008–2018. The mean precipitation is about 0.15 mm/h at the topographical height of 0–100 m, increases to the peak around 350 m, and then decreases rapidly with the heights. In other words, the hourly precipitation amount is highest over the mountain slopes, slightly smaller over coastal and plain areas, and smallest over the mountainous areas with high topography.

**Figure 5:**
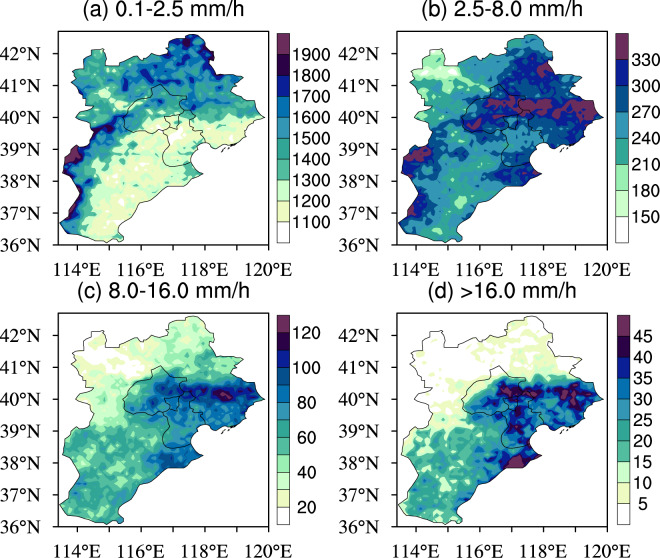
Distribution of cumulative hours of (**a**) light rain, (**b**) moderate rain, (**c**) heavy rain and (**d**) very heavy rain in the summer from 2008 to 2018 in the BTH region. Figure was produced using NCL V6.4.0 (http://www.ncl.ucar.edu/).

As indicated in Fig. 5, light rain is mainly concentrated in mountainous areas of the BTH region while moderate-to-very heavy rain occurs over the remaining areas. Especially, the highest frequencies of moderate, heavy and very heavy rain mainly appear over the south slope of the Yanshan Mountain.

**Figure 6:**
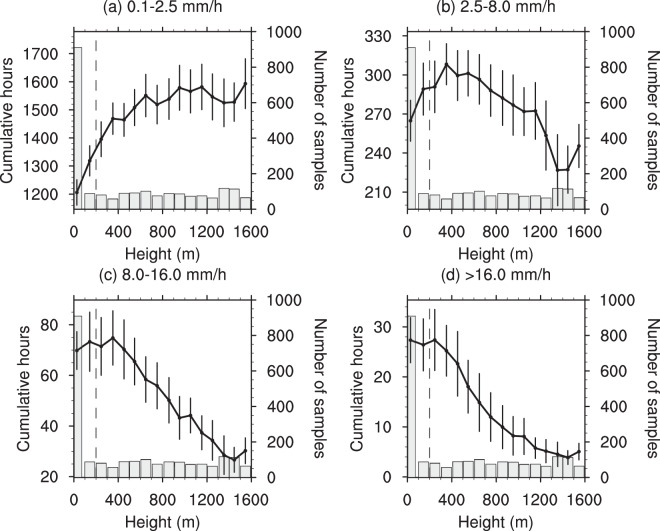
The linkages of cumulative hours of (**a**) light rain, (**b**) moderate rain, (**c**) heavy rain and (**d**) very heavy rain to topographical height (below 1600 m) in the BTH region for 2008–2018. The lines represent summer precipitation variations with topographical height (binned at an interval of 100 m), and the error bars are also shown (±0.5 standard deviation). The vertical blue dotted line in the map represents the boundary line between the plain and mountainous areas, and the column chart shows the number of samples in the corresponding set.

Figure 6 shows that cumulative hours of light rain, moderate rain, heavy rain and very heavy rain are all nonlinearly linked to the topographical conditions (below 1600 meters) in the BTH region for 2008–2018. Generally speaking, light rain frequency increases with the topographical heights while the frequencies of heavy and very heavy rain decrease. In comparison, moderate rain frequencies have a much more complex relationship with the topographical conditions.

## Discussion

Based on the newly emerging 0.1° high resolution hourly precipitation data from CMA for 2008–2018, this study features detailed spatial and diurnal characteristics of summer precipitation in the BTH region, and identified their close and nonlinear relationships with specific topographical conditions.

**Figure 7:**
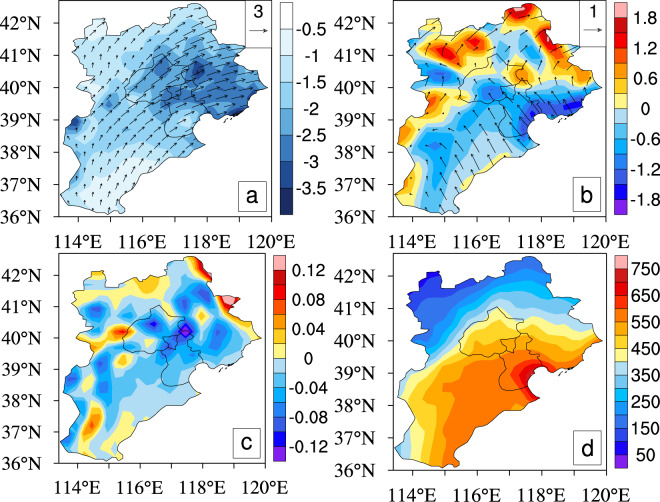
Climatic patterns of (**a**) 850 hPa wind field (vector, units: *m*/*s*) and vertically integrated moisture divergence (shaded, units: 10^−5^ *kg* · *m*^−2^ · *s*^−1^), (**b**) 10 m wind field (vector, units: *m*/*s*) and its divergence (shaded, units: 10^−5^ *s*^−1^), (**c**) 850 hPa vertical velocity (units: *Pa*/*s*), (**d**) convective available potential energy (CAPE) (units: *J*/*kg*) in the BTH region for the summers of 2008–2018. Figure was produced using NCL V6.4.0 (http://www.ncl.ucar.edu/).

What are the underlying physical processes explaining the close associations between summer precipitation and the topography over the BTH region? During the summer, the humid monsoon provides the main moisture source of precipitation over the BTH region (Fig. 7a). Additionally, near-surface southeasterly flow transports the moisture from the Bohai Sea to the BTH region (Fig. 7b). The strongest moisture convergence, the highest upward motion and the relatively large convective available potential energy (CAPE) together tend to cause the strongest summer precipitation over the south slope of the Yanshan Mountain (Figs. 1c and 7), in line with the windward slope effects^13,14^. And, favorable moisture, vertical motion and instable energy conditions may largely explain the strong summer precipitation over the coastal and plain areas and the east slope of Taihang Mountain.

Diurnal evolutions of summer wind flows, moisture divergence/convergence, vertical motion, and CAPE are further analyzed (see Supplementary Figs. S2–S5). The results show that diurnal evolutions of these physical factors generally correspond well with those of summer precipitation over the BTH region. Under the background of the humid summer monsoon, the mountain-valley breeze and the sea-land breeze largely modulate the spatial structure and diurnal evolutions of summer precipitation over the BTH region via their impacts on local atmospheric circulation, moisture transports, vertical motion, and so on.

In addition to the topography, land surface conditions such as soil type^33^, soil moisture^34,35^, soil temperature^36^ and the vegetation^37^ can modulate diurnal precipitation variations in the BTH region via their effects on surface energy and water fluxes, the atmospheric boundary layer, and local-to-regional atmospheric circulations. As the man-made land surface, the cities such as Beijing and Tianjin may have a certain impact on diurnal precipitation variations over urban areas, surrounding areas, and even the whole region^16,17,38–41^.

Based on previous studies, other processes such as aerosol-cloud interactions^42,43^ and large-scale climate patterns^44–49^ are proposed to affect summer precipitation in the BTH region. These complex physical mechanisms involved should be further explored by combing statistical analysis and model simulations in the future.

## Methods

### Data

The hourly precipitation data at a resolution of 0.1° over China for 2008–2018 were provided by the National Meteorological Information Center of China Meteorological Administration (NMIC-CMA) (http://data.cma.cn/). This gridded data were produced by the two-step data fusion algorithm of probability density function and optimal interpolation (PDF + OI) using the hourly precipitation observations at 30,000–40,000 automatic weather stations (AWSs) and the CMORPH^32^ dataset. The hourly high-resolution precipitation dataset newly developed by CMA based on the observations from very dense AWSs and the widely-used CMORPH dataset^50–53^ is applied to improve our understanding of diurnal precipitation variations over the BTH region.

For all summers (June-August) from 2008 to 2018, there are 776 missing hourly precipitation data over the BTH region (see Supplementary Table S1). The missing rate of the data is very small, and we remove these days which have any missing data in this study.

Additionally, this study also uses the gridded population data^54^ provided by the Resource and Environmental Science Data Center of the Chinese Academy of Sciences (RESDC-CAS) with a resolution of 1 km, and GTOPO30 topographical height data^55^ provided by the US Geological Survey (USGS) with a resolution of 30 seconds (about 1 km). Besides, hourly 850 hPa and 10 m wind flows, 850 hPa vertical velocity, vertically integrated moisture divergence and convective available potential energy (CAPE) at a resolution of 0.25° from ERA5 reanalysis dataset^56^ were also used to examine the underlying physical processes. The population and topographical height data are re-gridded onto the same resolution of hourly precipitation.

### Definition and analysis

A precipitation hour is defined as the hourly precipitation amount is greater than or equal to 0.1 mm/h. The hourly precipitation is further classified as light rain, moderate rain, heavy rain and very heavy rain based on the CMA and CAAC (Civil Aviation Administration of China) guideline (Table 1). The topography is divided into three categories: coastal (<10 m), plain (10–200 m), and mountain (>200 m) areas by taking into account the terrain characteristics of the BTH region.

**Table 1:** Classification of precipitation levels.

	Light rain	Moderate rain	Heavy rain	Very heavy rain
Hourly precipitation (mm/h)	0.1–2.5	2.5–8.0	8.0–16.0	>16.0

First, the 2008–2018 summer mean precipitation values are calculated to investigate detailed spatial precipitation features and their correspondences to topographical conditions and population distribution over the BTH region (Fig. 1). Second, we perform the analysis on diurnal evolutions of summer precipitation for 2008–2018, and also the spatial distribution of the precipitation peaks in the BTH region (Figs. 2, 3 and S1). Third, we evaluate the relations of summer mean precipitation, light-to-very heavy precipitation frequencies to the topographical heights in the BTH region (Figs. 4–6). Last, we analyze the underlying physical processes responsible for the linkages between summer precipitation and the topography (Figs. 7 and S2–S5).

## Supplementary information

Supplementary Information.
