# Supplementary material for: Diurnal Variations of Summer Precipitation Linking to the Topographical Conditions over the Beijing-Tianjin-Hebei Region
Source: Sci Rep. 2020 Jun 16;10:9701. doi: 10.1038/s41598-020-65743-1 (PMC7298024; doi:10.1038/s41598-020-65743-1)
Supplement: Supplementary file 1 — Supplementary Information. [file 41598_2020_65743_MOESM1_ESM.docx]

**Supplementary Information for**

**Diurnal Variations of Summer Precipitation Linking to the Topographical Conditions over the Beijing-Tianjin-Hebei Region**

Ziyi Song^1,2^&Jingyong Zhang^2,3^

^1^Key Laboratory of Meteorological Disaster, Ministry of Education (KLME)/Collaborative Innovation Center on Forecast and Evaluation of Meteorological Disasters (CIC-FEMD), Nanjing University of Information Science and Technology, Nanjing, 210044, China

^2^Center for Monsoon System Research, Institute of Atmospheric Physics, Chinese

Academy of Sciences, Beijing, 100029, China

^3^ College of Earth and Planetary Sciences, University of Chinese Academy of Sciences, Beijing 100049, China

Corresponding author: Prof. Jingyong Zhang, Center for Monsoon System Research, Institute of Atmospheric Physics, Chinese Academy of Sciences, Beijing, 100029, China. Email: [zjy@mail.iap.ac.cn](mailto:zjy@mail.iap.ac.cn).

This file includes:

**Table S1**

**Figures S1-S5**

**Table S1.** Time of missing precipitation data

| Year | Missing times |
| --- | --- |
| 2013 | 09:00 on 6 August |
| 2014 | 18:00,21:00,22:00 on 29 June and 0:00,1:00,2:00,3:00 on 30 June |
| 2016 | 8:00 on 21 June-8:00 on 22 June, 8:00 on 9 August-8:00 on 10 August |
| 2017 | 8:00 to 8:00 the next day on 1, 2, 3, 16, 17, 22, 23, 24, 30 June  8:00 to 8:00 the next day on 1,7,8,14,15,21,22,28,29July  8:00 to 8:00 the next day on 3,4,5,11,12,17,18,19,24,25,26,31August |


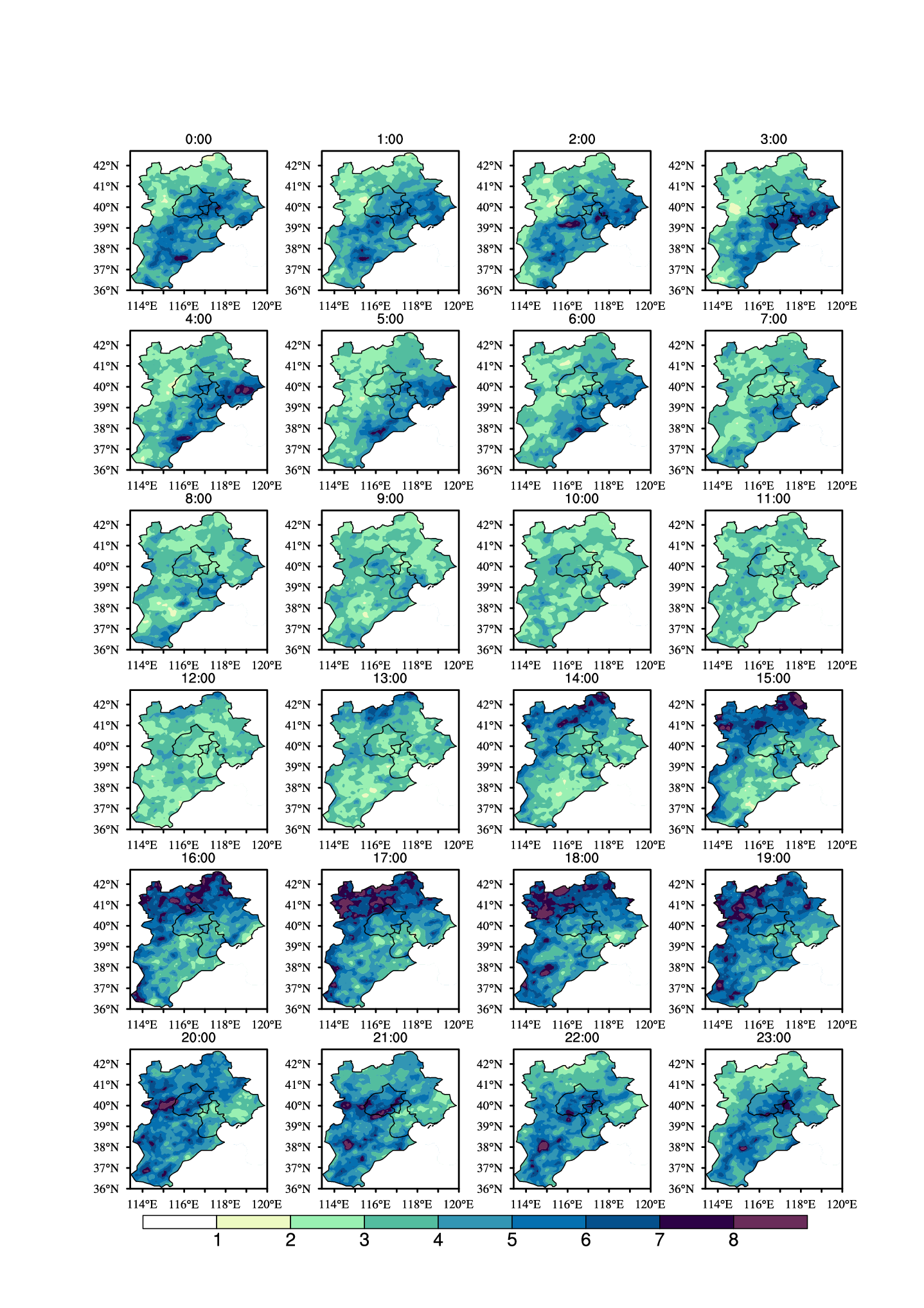


**Figure S1.** The percentage of hourly precipitation to 24-hours cumulative precipitation in the Beijing-Tianjin-Hebei region of summer for 2008-2018 (units: %). Figure was produced using NCL V6.4.0 (<http://www.ncl.ucar.edu/>).


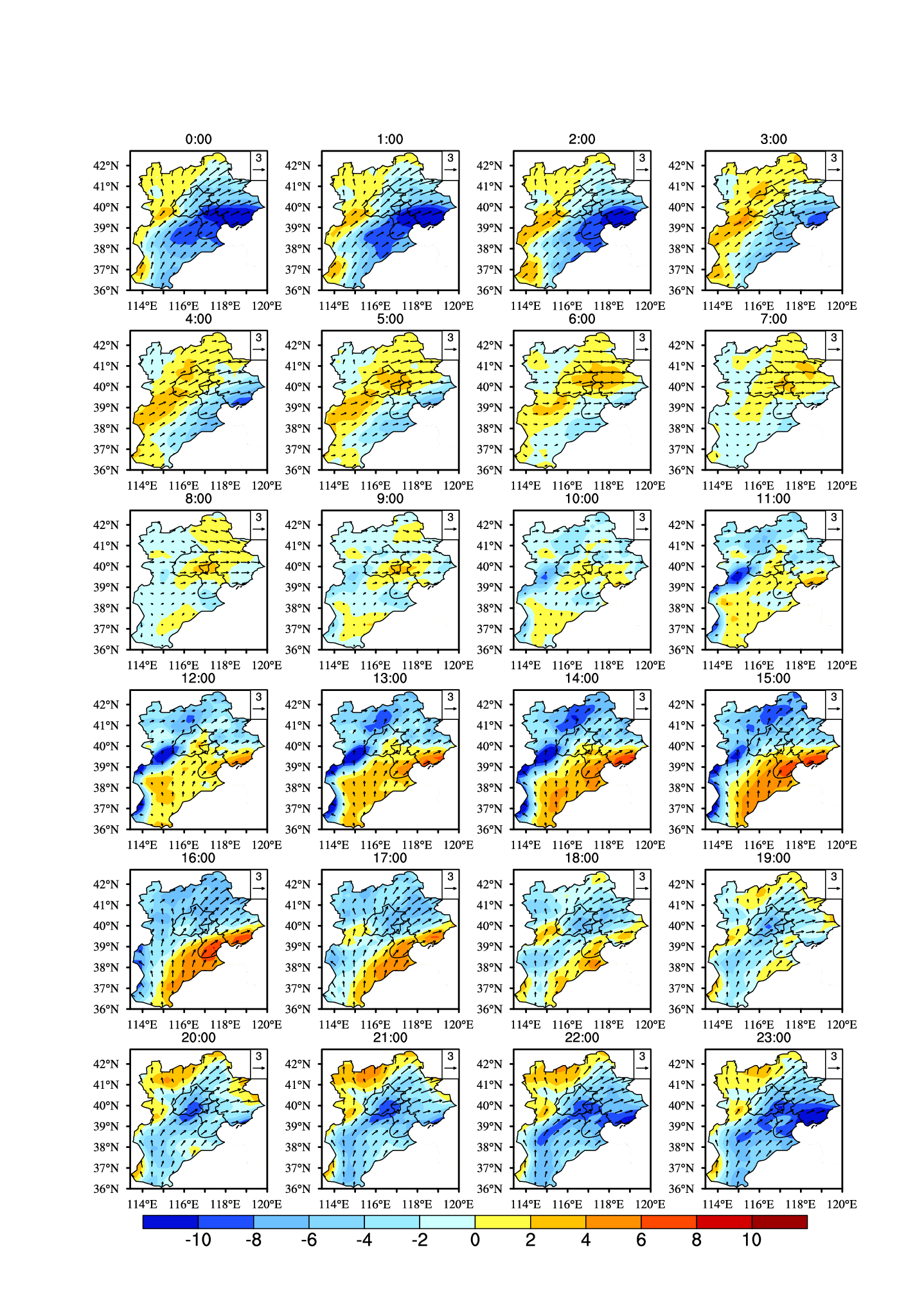


**Figure S2.** The diurnal variation of 850hPa wind field (vector, units: $m/s$) and vertically integrated moisture divergence (shaded, units: ${10}^{-5}kg\cdot m^{-2}\cdot s^{-1}$) in the Beijing-Tianjin-Hebei region for the summers of 2008-2018. Figure was produced using NCL V6.4.0 (<http://www.ncl.ucar.edu/>).


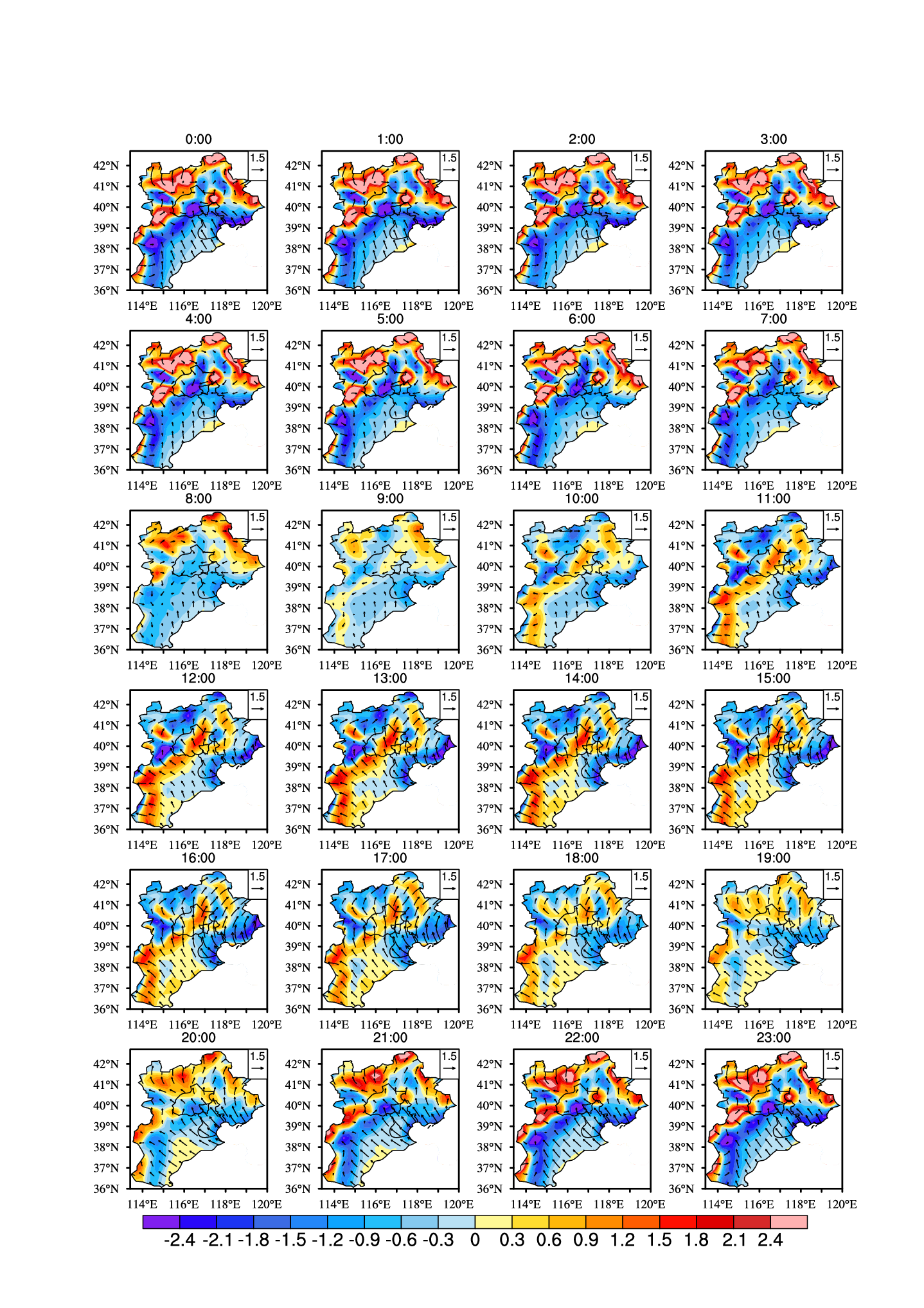


**Figure S3.** The diurnal variation of 10m wind field (vector, units: $m/s$) and its divergence (shaded, units: ${10}^{-5}s^{-1}$) in the Beijing-Tianjin-Hebei region for the summers of 2008-2018. Figure was produced using NCL V6.4.0 (<http://www.ncl.ucar.edu/>).


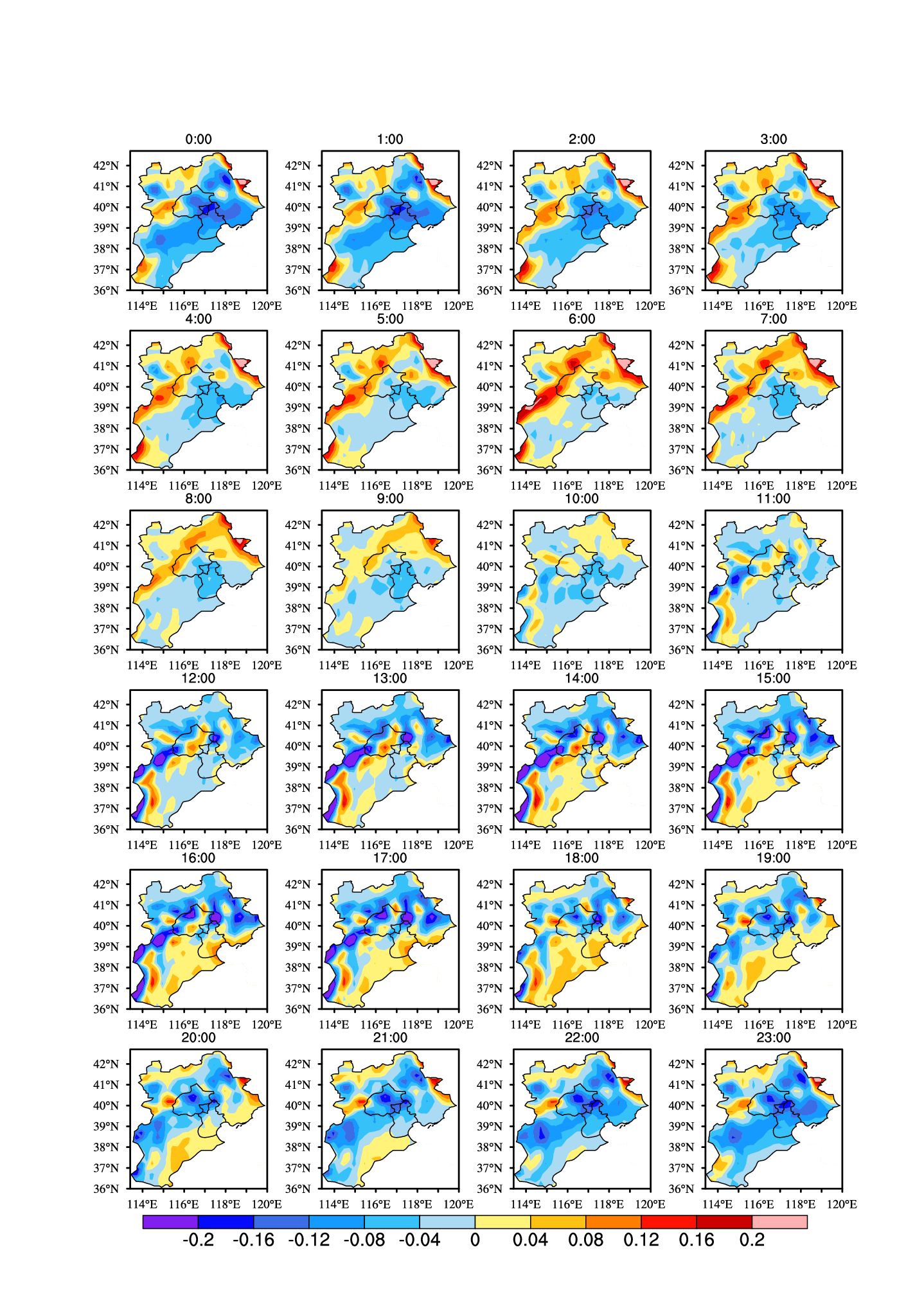


**Figure S4.** The diurnal variation of 850hPa vertical velocity (units: $Pa/s$) in the Beijing-Tianjin-Hebei region for the summers of 2008-2018. Figure was produced using NCL V6.4.0 (<http://www.ncl.ucar.edu/>).


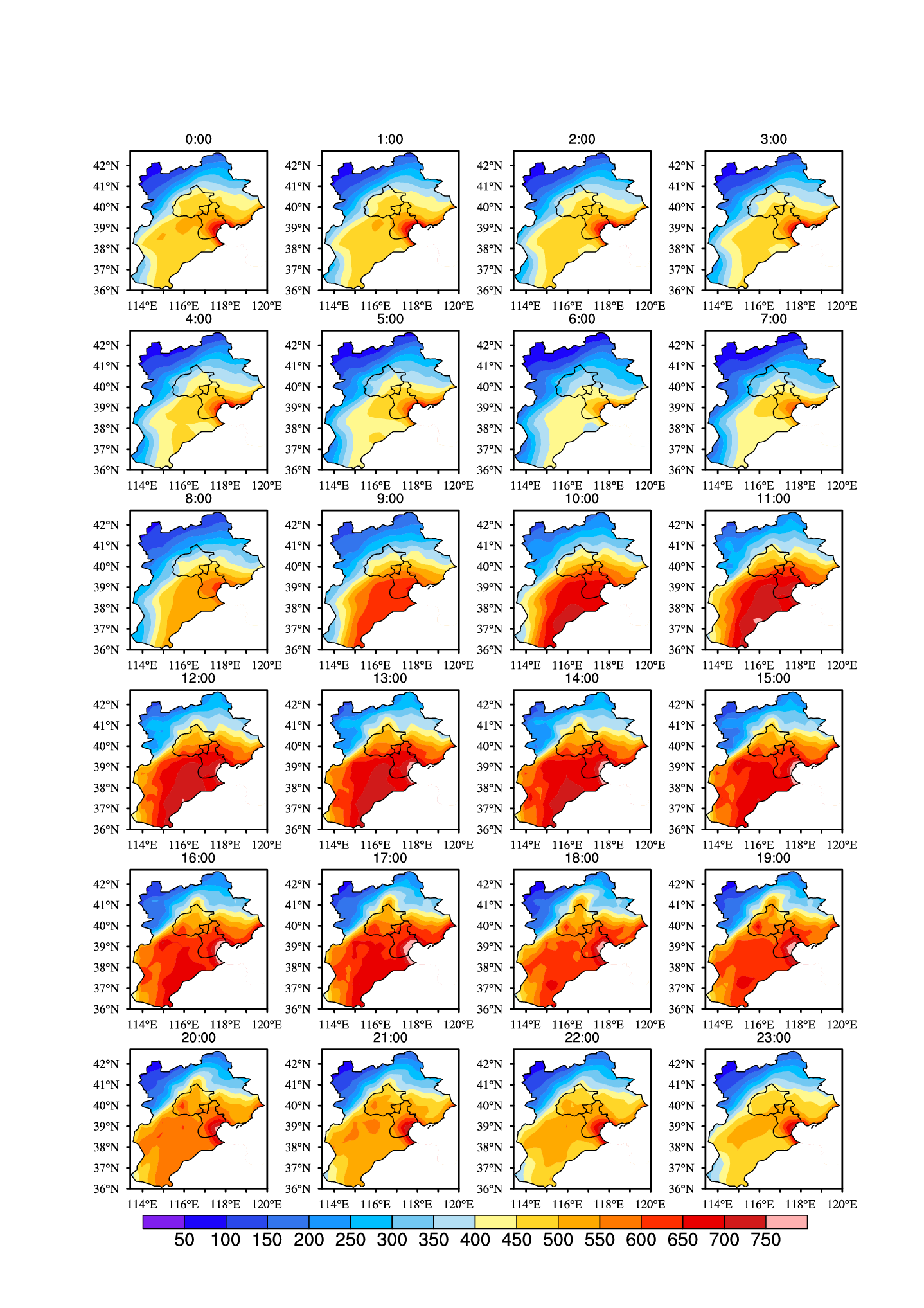


**Figure S5.** The diurnal variation of convective available potential energy (CAPE) (units: $J/kg$) in the Beijing-Tianjin-Hebei region for the summers of 2008-2018. Figure was produced using NCL V6.4.0 (<http://www.ncl.ucar.edu/>).
